# Prediction of major outcomes in patients with malignant hypertension using machine learning: A report from the West Birmingham malignant hypertension registry

**DOI:** 10.1111/eci.70052

**Published:** 2025-04-18

**Authors:** Antonios A. Argyris, Hironori Ishiguchi, Yang Chen, Yalin Zheng, Alena Shantsila, Eduard Shantsila, D. Gareth Beevers, Gregory Y. H. Lip

**Affiliations:** ^1^ Liverpool Centre for Cardiovascular Science University of Liverpool, Liverpool John Moores University and Liverpool Heart and Chest Hospital Liverpool UK; ^2^ University Department of Medicine City Hospital Birmingham UK; ^3^ Danish Center for Health Services Research, Department of Clinical Medicine Aalborg University Aalborg Denmark; ^4^ Medical University of Bialystok Bialystok Poland

**Keywords:** all‐cause mortality, machine learning, malignant hypertension, outcomes

## Abstract

**Background:**

Malignant hypertension (MHT) is a rare, yet severe condition with high morbidity and mortality. We aimed to assess the potential of machine learning (ML) algorithms in forecasting prognostic outcomes in MHT patients.

**Methods:**

Data from the West Birmingham MHT Registry were used. We evaluated the efficacy of 9 ML algorithms, CatBoost, Decision Tree (DT), Light‐Gradient Boosting Machine (LightGBM), K‐Nearest Neighbours (KNN), Logistic Regression (LR), Multi‐Layer Perceptron (MLP), Random Forest (RF), Support Vector Machine (SVM) and XGBoost in predicting a composite outcome of all‐cause mortality/dialysis. Evaluation metrics included the area under the receiver operating characteristic curve (AUC) and F1 score. SHapley Additive exPlanations values were employed to quantify the importance of each feature.

**Results:**

The cohort comprised 385 individuals with MHT (mean age 48 ± 13 years, 66% male). During a median follow‐up of 11 (interquartile range: 3–18) years, 282 patients (73%) experienced the composite outcome. Among 24 demographic and clinical variables, 16 were selected into the ML models. The SVM, LR, and MLP models exhibited robust predictive performance, achieving AUCs of .81 (95% CI: .70–.90), .82 (95% CI: .71–.92) and .81 (95% CI: .71–.90), respectively. Furthermore, these models demonstrated high F1 scores (SVM: .75, LR: .80. MLP: .75). Age, smoking, follow‐up systolic blood pressure, and baseline creatinine were commonly identified as primary prognostic features in both SVM and LR models.

**Conclusions:**

The application of ML algorithms facilitates effective prediction of prognostic outcomes in MHT patients, illustrating their potential utility in clinical decision‐making through more targeted risk stratification and individualised patient care.

## Graphical abstract

Malignant hypertension (MHT) is a rare, severe condition with high morbidity and mortality. Using data from 385 patients in the West Birmingham MHT Registry, this study evaluated nine machine learning (ML) algorithms to predict a composite outcome of all‐cause mortality/dialysis over a median 11‐year follow‐up. Logistic Regression (AUC: .82), Support Vector Machine (AUC: .81), and Multi‐Layer Perceptron (AUC: .81) demonstrated robust predictive performance. Age, smoking, systolic blood pressure, and baseline creatinine emerged as key prognostic features. ML holds promise for improving MHT risk stratification.

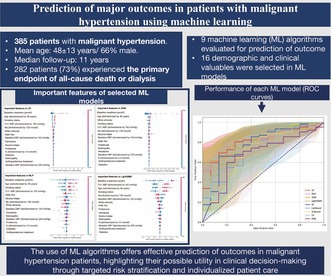

## 1 INTRODUCTION

Malignant hypertension (MHT), characterized by severely elevated blood pressure and rapid progression of organ damage, including the retina, the brain, and the kidneys, poses significant challenges in clinical management due to its high mortality rates and diverse underlying pathophysiology.^1^ Despite advances in hypertension management, MHT remains a significant cause of morbidity and mortality worldwide, while data on its prevalence remain scarce.^2^, ^3^, ^4^, ^5^, ^6^, ^7^, ^8^, ^9^, ^10^

Identifying the key prognostic factors that drive mortality in patients with MHT is paramount for informing treatment strategies and improving patient outcomes.^5^, ^7^, ^9^, ^11^, ^12^, ^13^ Traditional statistical models have provided valuable insights, yet the complex interplay of various clinical and demographic factors demands more sophisticated analytical approaches, emphasizing not only single risk factors but also multimorbidity aspects.

In recent years, machine learning (ML) techniques have emerged as powerful tools for uncovering intricate patterns within large and heterogeneous datasets, offering novel pathways for data analysis, predictive modelling and decision support, that are especially useful in the management of chronic cardiovascular disease (CVD).^14^, ^15^, ^16^, ^17^ Machine learning algorithms possess the ability to discern complex patterns and relationships by leveraging algorithms that iteratively learn from data, adjusting their parameters to improve performance on a specific task such as disease diagnosis, prognosis, treatment optimization, and outcome prediction.^18^, ^19^

The aim of the present analysis was (i) to unveil specific patient characteristics – including demographic, clinical and biochemical markers – that have a strong predictive ability for major adverse outcomes in a population with MHT, using machine learning techniques, (ii) to evaluate the performance of different machine learning models in predicting major adverse outcomes. For this purpose, we employed data from the West Birmingham Malignant Hypertension Registry.

## 2 METHODOLOGY

### 2.1 Data source and study population

The West Birmingham Malignant Hypertension registry consists of 460 patients who were diagnosed with MHT since 1958. This registry was established in 1977; all patients were followed up and data regarding demographic characteristics, cardiovascular risk factors, blood pressure (BP) measurements, and clinical outcomes were collected from the hospital register, patient files, hospital admissions, or primary care facilities.^2^, ^3^, ^5^ The registry used in this study was initiated in 1977, before formal research ethics committee structures were established in the United Kingdom. At that time, ethical approval was not required for registry creation. This study involves a secondary analysis of anonymized historical data, and therefore, no additional ethical approval is required under current ethical standards. The population is multiethnic, comprising 64% whites, 25% South Asians, and 11% Afro‐Caribbeans. The diagnosis of MHT was based on the presence of severe BP elevation at the baseline visit, along with the corresponding findings in the fundoscopic examination (retinal linear or flame‐shaped haemorrhages and/or exudates and/or cotton wool spots, with or without papilledema).^2^ BP was measured at baseline and during subsequent clinical visits according to standardized protocols, using validated brachial cuff sphygmomanometers. However, the exact timing of these follow‐up visits was not systematically recorded in the original dataset. We excluded patients with diseases that could accelerate progression to a worse clinical outcome, such as patients with glomerulonephritis, systemic lupus erythematosus, renal cell carcinoma, polycystic kidney disease, or systemic sclerosis. Patients with available data and known status until May 2016 were included.

### 2.2 Outcomes

The primary endpoint of the present study was to evaluate the efficacy of ML to predict the development of all‐cause death or dialysis. A secondary endpoint was to evaluate the efficacy of ML in predicting the development of each of the two components of the primary outcome (i.e. all‐cause death, dialysis). The secondary endpoint was also to identify key factors predictive of these outcomes. Data on outcomes were derived from hospital registers, patient files, hospital admissions or primary care facilities. The time of follow‐up was determined from the date of baseline visit until the date of event occurrence or until May 2016. The primary endpoint of all‐cause mortality and dialysis was chosen as it reflects critical and life‐threatening complications associated with MHT. These outcomes provide a comprehensive assessment of disease burden and progression in this high‐risk population, supported by reliably documented data within the dataset to ensure robust analyses. Furthermore, cardiovascular events and their associated risk factors have been extensively reported in a prior study,^7^ allowing this study to focus on other clinically significant endpoints.

### 2.3 Data pre‐processing

We collected variables about patient characteristics that included age, sex, ethnicity, body mass index (BMI), smoking status and alcohol intake, diabetes, antihypertensive treatment at baseline, retinopathy, urinalysis results (proteinuria and hematuria), urea levels, electrolytes (Na, K), systolic BP (SBP), diastolic BP (DBP) and pulse pressure (PP) measurement at 3 visit points (at baseline, the first follow‐up [FU1] and the second follow‐up [FU2] visit) and creatinine levels in 2 visit points (at baseline and FU1).

Outlier values were determined using a Z‐score threshold greater than ±3 for normally distributed data and for non‐normally distributed data, outlier values were defined as values more than three times the interquartile range (IQR) from the first or third quartile. The Miceforest program was employed for the imputation of these outlier values, as well as for other missing data, including categorical variables.

### 2.4 Feature selection

From the collecting dataset, variables with a missing rate exceeding 20% were excluded from the ML analysis to ensure data integrity. We assessed the inter‐variable correlations using Pearson correlation coefficients (*R*‐values) and Variance Inflation Factor (VIF) values. Continuous variables exhibiting *R*‐values greater than .6 or VIF values exceeding 10 were binarized based on their mean or median values to mitigate multicollinearity effects. Variables that still demonstrated high R or VIF values post‐binarization were omitted from the analysis. The remaining variables were then incorporated into the ML analysis.

### 2.5 Machine learning

We evaluated the efficacy of 9 different ML models in identifying patients at risk of the primary endpoint (all‐cause mortality/dialysis). The models analysed included CatBoost, Decision Tree (DT), Light Gradient Boosting Machine (LightGBM), K‐Nearest Neighbours (KNN), Logistic Regression (LR), Multi‐Layer Perceptron (MLP), Random Forest (RF), Support Vector Machine (SVM) and XGBoost. In the analysis of the secondary endpoint, we utilised the three models with the highest performance.

We divided the cohort into training (80%) and test (20%) sets. Each model was trained using hyperparameters that were fine‐tuned based on grid search outcomes and validated through five‐fold cross‐validation. After determining the optimal hyperparameters for each model, we assessed the significance of each feature using SHapley Additive exPlanations (SHAP) values. In each model, the feature exhibiting the highest SHAP value was designated as the key feature.

### 2.6 Statistical analysis

Normally distributed continuous variables were expressed as mean ± SD and non‐normally distributed as median and IQR. The t‐test or the Mann–Whitney *U* test was facilitated according to the distribution of these variables. Categorical variables were expressed as counts and percentages. The Fisher test was used to compare these data. Receiver operating characteristics (ROC) curves were plotted to contrast the performance of the models. The efficacy of each ML model was gauged by the area under the ROC curve (AUC), inclusive of a 95% confidence interval (CI), alongside the optimal threshold determined by the Youden index applied across various metrics. Additional performance metrics included precision (ratio of true positives to all positive predictions, in other words: positive predictive value), recall (ratio of true positives to all actual positives, in other words: sensitivity), F1 score (the harmonic mean of precision and recall), accuracy (proportion of true results, both positive and negative, in the population), negative predictive value (ratio of true negatives to all negative predictions), positive likelihood ratio (ratio of sensitivity to 1‐specificity), negative likelihood ratio (ratio of 1‐sensitivity to specificity) and specificity. Statistical analysis was performed in R version 4.0.4, while programming tasks were completed using Python 3.10.12. A two‐sided *p* < .05 was considered statistically significant.

## 3 RESULTS

### 3.1 Patient characteristics

Within the total cohort of 460 patients, 385 were confirmed to have information on all‐cause mortality/dialysis (Figure S1). Among these, 282 patients (73%) experienced the outcome over a median follow‐up of 11 years (IQR: 3–18 years), while 260 and 39 developed all‐cause mortality and dialysis, respectively. Patient demographic characteristics are shown in Table 1. Those who encountered all‐cause mortality/dialysis were notably older, had a significantly higher smoking prevalence, and alcohol consumption.

**TABLE 1:** Patient characteristics presented based on the presence or absence of the outcome.

Variables	All‐cause mortality/dialysis (*n* = 282)	Patients alive and not on dialysis (*n* = 103)	*p* Value
Age, years [0]	50 ± 13	42 ± 11	<.001
Male Sex, *n* (%) [0]	179 (63)	74 (72)	.16
Ethnicity, white, *n* (%) [.3]	183 (65)	51 (50)	<.001
BMI, kg/m^2^ [49]	27.3 ± 5.7	29.4 ± 6.4	<.001
Smoking, *n* (%) [6]	157 (56)	29 (28)	<.001
Alcohol intake (units/week) [14]	2 (0, 14)	2 (0, 8.5)	<.001
Diabetes, *n* (%) [.6]	13 (4.6)	5 (4.8)	.75
Antihypertensive treatment, *n* (%) [3]	86 (30)	22 (21)	.07
Retinopathy, *n* (%) [0]	198 (70)	62 (60)	.08
Proteinuria, *n* (%) [8]	191 (68)	62 (60)	.1
Hematuria, *n* (%) [8]	59 (21)	27 (26)	.15
Baseline SBP, mmHg [1]	229 ± 28	230 ± 31	<.001
Baseline DBP, mmHg [1]	141 ± 20	144 ± 19	<.001
Baseline PP, mmHg [1]	87 ± 25	86 ± 26	<.001
Baseline Urea, mmol/l [4]	10 (7, 18)	7 (5, 8)	<.001
Na, mmol/l [5]	138 ± 4.8	140 ± 3.3	<.001
K, mmol/l [5]	4.0 ± .8	3.9 ± .7	<.001
FU1 SBP, mmHg [9]	170 ± 32	140 ± 16	<.001
FU1 DBP, mmHg [9]	101 ± 19	87 ± 11	<.001
FU1 PP, mmHg [9]	69 ± 22	53 ± 13	<.001
FU2 SBP, mmHg [8]	177 ± 35	149 ± 24	<.001
FU2 DBP, mmHg [8]	106 ± 22	94 ± 17	<.001
FU2 PP, mmHg [8]	71 ± 22	55 ± 13	<.001
Baseline creatinine, μmol/l [11]	161 (117, 343)	125 (96, 170)	<.001
FU1 creatinine, μmol/l [29]	192 (127, 469)	119 (94, 150)	<.001

*Note*: Continuous variables are presented as mean ± standard deviation or median (first quartile, third quartile). Categorical variables are presented as number and frequencies as appropriate. [] indicates missing rate (%).

Abbreviations: BMI, body mass index; DBP, diastolic blood pressure; FU1, first follow up visit; FU2, second follow up; PP, pulse pressure; SBP, systolic blood pressure.

Even though both groups exhibited clinically non‐significant differences of BP measurements at baseline, we noticed statistically and clinically higher levels of BP in the group that encountered the outcome at both follow‐up visits (SBP 170 ± 32 mm Hg vs. 140 ± 16 mm Hg, *p* < .001, respectively; DBP 101 ± 19 mm Hg vs. 87 ± 11 mm Hg, *p* < .001, respectively, at the FU1). Furthermore, creatinine levels at both visit points were significantly elevated in patients who developed the outcome.

### 3.2 Feature selection

From the total of 24 variables in patient characteristics, we refined the selection for use in constructing the ML models. Initially, BMI (with 49% missing data) and creatinine levels at FU1 (with 29% missing data) were removed due to their high rates of missing values. Variables such as PP at baseline, DBP at FU1, PP at FU1, urea levels, SBP at FU2, DBP at FU2 and PP at FU2 were also excluded because of their high *R*‐values, even after dichotomization. Consequently, 16 variables were retained as features for the models. Due to elevated VIF values, all continuous variables, with the exception of alcohol intake and baseline creatinine, were dichotomized based on their mean or median values. After these preprocesses, all the variables had satisfactory VIF scores and *R*‐values (Figure S2, Table S1).

### 3.3 Performance of each machine learning model

**FIGURE 1:**
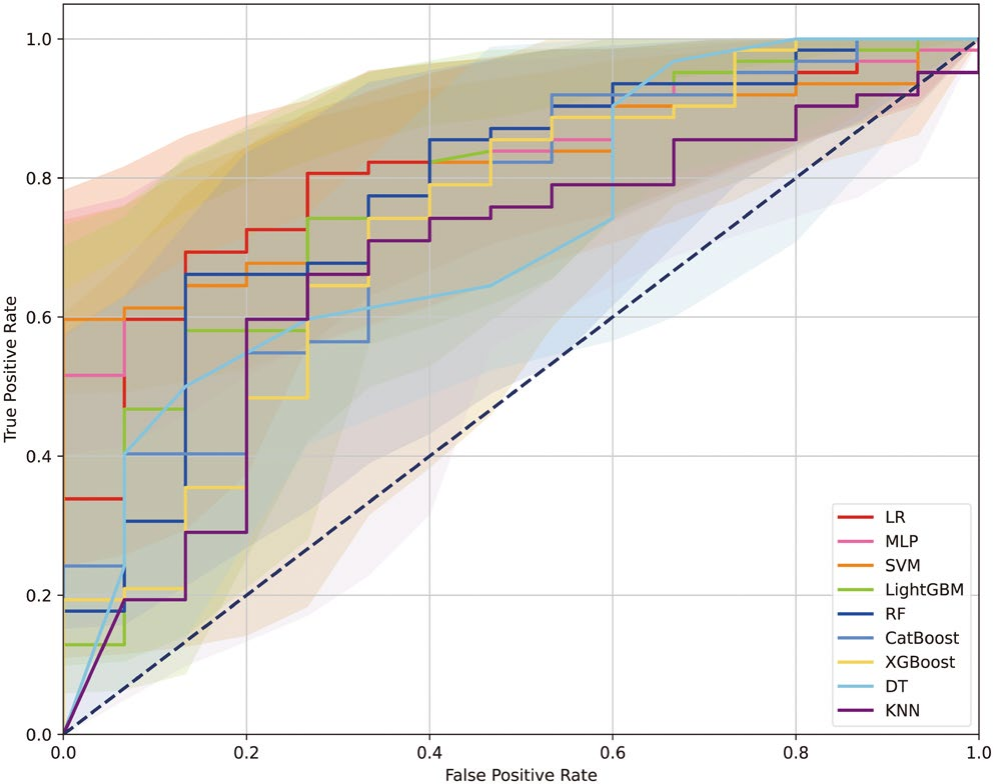
Presentation of the receiver operating characteristic curves across machine learning models for the primary endpoint. Each curve was delineated as AUC with 95% CI. AUC, area under the curve; CI, confidence interval; DT, decision tree; GBM, gradient boosting machine; KNN, K‐nearest neighbours; LR, logistic regression; MLP, multi‐layer perceptron; RF, random forest; SVM, support vector machine.

The performance metrics for each ML model are presented in Table 2, while the optimal hyperparameters determined via grid search are shown in Table S2. The comparative analysis of the models is depicted in Figure 1. The LR model, the LightGBM model, the CatBoost model and the XGBoost model showed satisfactory F1 scores (>.8). Notably, the LR model (AUC: .82, 95% CI: .71–.92), the MLP model (AUC: .81, 95% CI: .71–.91) and the SVM model (AUC: .81, 95% CI: .70–.90) demonstrated superior AUC values (>.8) in comparison to other models. The identified optimal thresholds varied between .64 and .86.

**TABLE 2:** Presentation of the performance of different machine learning models for the primary outcome.

Models	AUC	95%CI	Optimal threshold	Precision (PPV)	Recall (sensitivity)	F1 Score	Accuracy	NPV	Positive likelihood ratio	Negative likelihood ratio	Specificity
LR	.82	.71–.92	.71	.96	.69	.80	.73	.83	5.31	.36	.87
MLP	.81	.71–.90	.88	.97	.61	.75	.68	.81	8.71	.42	.93
SVM	.81	.70–.90	.75	1.0	.60	.75	.68	.80	–	.40	1.0
LightGBM	.78	.64–.91	.70	.92	.74	.82	.74	.84	2.74	.36	.73
RF	.78	.64–.91	.73	.95	.66	.78	.70	.81	5.08	.39	.87
CatBoost	.74	.61–.89	.6	.89	.79	.84	.75	.87	1.98	.35	.60
XGBoost	.73	.57–.88	.75	.90	.74	.81	.73	.83	2.24	.35	.67
DT	.72	.57–.87	.75	.94	.50	.65	.57	.71	3.85	.57	.87
KNN	.67	.53–.82	.69	.93	.6	.73	.64	.79	3.00	.50	.80

Abbreviations: AUC, area under the curve; CI, confidence interval; DT, decision tree; GBM, gradient boosting machine; KNN, K‐nearest neighbours; LR, logistic regression; MLP, multi‐layer perceptron; NPV, negative predictive value; PPV, positive predictive value; RF, random forest; SVM, support vector machine.

### 3.4 Key factors

SHAP values facilitated the comparison of feature contributions across the various ML models. Among the models, 7 models including those with notably high AUC values – particularly the LR, MLP and SVM models – consistently highlighted baseline creatinine levels as the key feature, as evidenced by the highest SHAP values (Figure S3). For other notable features, SBP at FU1 was highlighted as a key feature exclusively by the CatBoost model and ranked as the second most influential feature in both LightGBM and RF models. Additionally, age was recognized as a significant predictor across several models, being a key feature in DT and the second most influential in LR, MLP, SVM, CatBoost and XGBoost. Furthermore, the high negative SHAP values for covariates such as Na, DBP, male sex and proteinuria suggest that these features are associated with reduced predicted risks of the outcomes in the studied models. For example, normal or higher sodium levels and DBP may indicate better physiological stability, while lower proteinuria levels could reflect less severe renal dysfunction. In addition, male sex might exhibit a lower observed risk in this dataset, contributing negatively to the predictions.

### 3.5 Prediction of secondary endpoints

**FIGURE 2:**
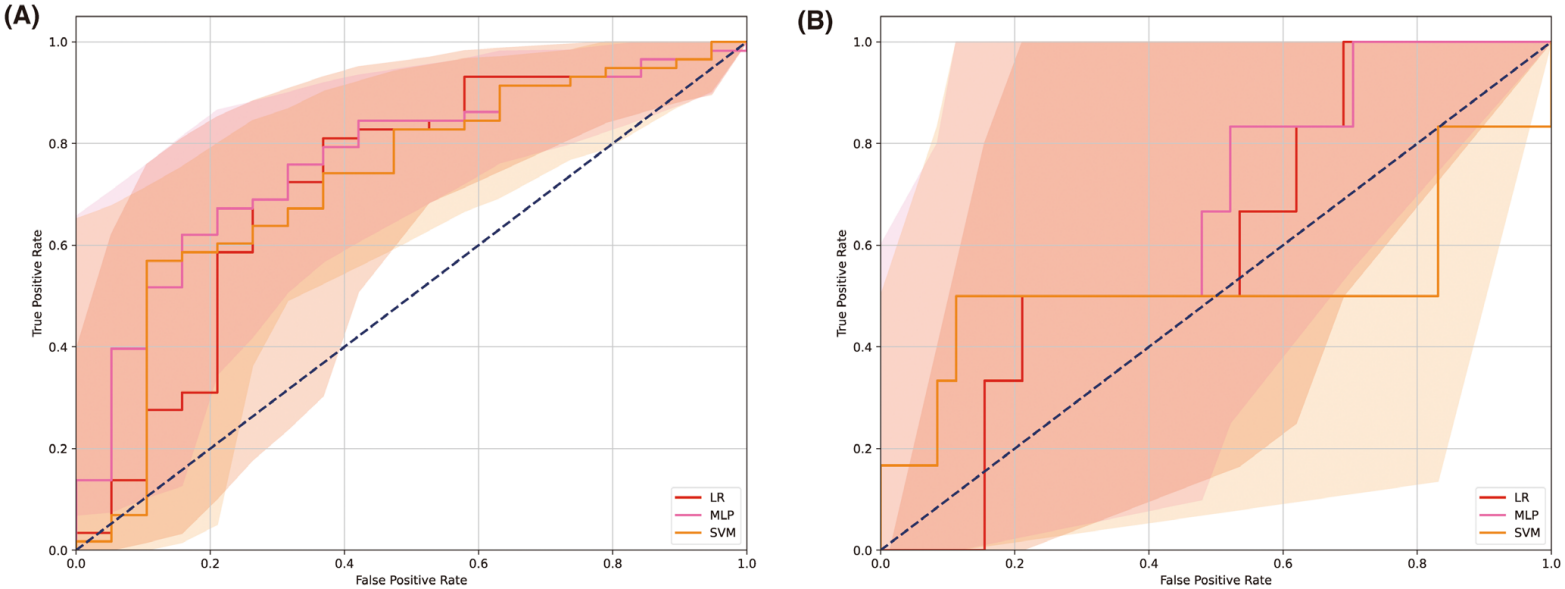
Presentation of the receiver operating characteristic curves across machine learning models for secondary endpoints. (A) Prediction for all‐cause mortality. (B) Prediction for dialysis. Each curve was delineated as AUC with 95% CI. AUC, area under the curve; CI, confidence interval; LR, logistic regression; MLP, multi‐layer perceptron; SVM, support vector machine.

Based on the primary endpoint results, LR, MLP and SVM were selected for the analysis of secondary endpoints. The performance metrics for these secondary endpoints within each ML model are detailed in Table 3, with the optimal hyperparameters determined via grid search provided in Table S3. In the case of all‐cause mortality, the AUCs were consistent across the 3 models (Figure 2A, all showing .71). Regarding key factors identified through SHAP values, LR and MLP models detected age as the crucial feature, whereas SVM highlighted baseline creatinine levels (Figure S4). For dialysis outcomes, despite the challenges posed by an unbalanced population (with only 10% of patients experiencing events), LR and SVM models demonstrated satisfactory AUCs (Figure 2B, at .70 and .69, respectively). These 2 models identified age and baseline creatinine levels as key predictors, mirroring findings from other endpoints, while highlighting also the contribution of alcohol consumption and the presence of various forms of renal damage, including proteinuria and hematuria (Figure S5).

**TABLE 3:** Presentation of the performance of different machine learning models for the secondary outcomes (all‐cause mortality, dialysis).

Models	AUC	95%CI	Optimal threshold	Precision (PPV)	Recall (sensitivity)	F1 Score	Accuracy	NPV	Positive likelihood ratio	Negative likelihood ratio	Specificity
Predicting all‐cause mortality											
LR	.71	.56–.86	.65	.88	.66	.75	.68	.66	2.54	.46	.74
MLP	.71	.56–.86	.54	.87	.81	.84	.77	.76	2.19	.30	.63
SVM	.71	.57–.84	.66	.91	.55	.69	.62	.61	3.44	.54	.84
Predicting dialysis											
LR	.70	.5–.92	.41	.13	1.0	.22	.46	.46	1.69	0	.41
MLP	.54	.28–.84	.03	.1	.83	.18	.40	.43	1.32	.46	.37
SVM	.69	.50–.84	.10	.18	.83	.29	.69	.69	2.59	.25	.68

Abbreviations: AUC, area under the curve; CI, confidence interval; LR, logistic regression; MLP, multi‐layer perceptron; NPV, negative predictive value; PPV, positive predictive value; SVM, support vector machine.

## 4 DISCUSSION

This is the first study to examine the prognostic role of different patient characteristics, evaluated in a cluster manner, in the development of all‐cause mortality/dialysis in a population with MHT using a variety of machine learning models. We have demonstrated that – among 9 ML algorithms trained – 3 models, namely LR, MLP and SVM, performed best to discriminate patients at high risk of developing the major adverse outcome, identifying key prognostic features between 16 different characteristics. Moreover, significant predictors for each of the secondary endpoints were highlighted.

In recent years, the role of ML has emerged as an important and useful tool in the field of cardiovascular diseases. This is especially evident in complex clinical entities, such as atrial fibrillation, stroke or cardiovascular risk management in diabetes and liver diseases,^14^, ^15^, ^16^, ^17^, ^18^, ^20^, ^21^ where different patterns of risk modifiers and risk scores have been unveiled.^22^, ^23^

The present study evaluated the performance of various ML models in predicting the composite outcome. Notably, LR, MLP and SVM models demonstrated superior AUC values (>.8), suggesting their potential utility in prognosticating all‐cause mortality or dialysis requirement in patients with MHT. Moreover, the LightGBM model, the CatBoost model, and the XGBoost model showed satisfactory F1 scores (>.8), despite the AUCs being moderate (>.7). In summary, the LR model achieved a fair balance between high AUC and F1 score compared to all other models. In our study, we employed different metrics to assess the performance of the different algorithms. While AUC provides an overall measure of the model's discriminative ability, precision and the F1 score offer insights into its performance on positive class predictions, emphasizing the balance between accurate positive predictions and the ability to capture all positive instances. Accuracy, on the other hand, offers a holistic view of the model's correctness across both positive and negative classes but may be less informative in imbalanced datasets. Sensitivity and specificity provide additional granularity by focusing on the model's ability to correctly identify positive and negative instances, respectively. The choice of which metric to prioritise depends on the relative costs of false positives and false negatives in the specific application. Ultimately, a comprehensive evaluation of the model should consider multiple metrics to assess its overall value and suitability for the prognostic task, ensuring a nuanced understanding of its strengths and limitations.

Furthermore, for the secondary endpoint (dialysis), we recognise that the relatively low F1 scores and accuracy values of the LR and SVM models may be due in part to the inherent category imbalance in the dataset, which tends to bias model predictions towards the majority of categories. While the AUC values reflect an acceptable level of discrimination, these metrics alone may not reflect the model's ability to balance false positives and false negatives for this endpoint. Future work may employ resampling techniques (e.g. Synthetic Minority Over‐sampling Technique) to improve classification performance. In addition, exploring advanced deep learning models (e.g. Convolutional Neural Networks) or integrating multi‐model frameworks (e.g. Stacking Ensemble Learning Technique) may help to address the complex interactions between clinical variables and improve predictive accuracy and clinical applicability. In addition, external validation of larger sample independent cohorts is required.

Feature importance analysis using SHAP values revealed consistent patterns across different ML models. Among the models, baseline creatinine levels frequently emerged as the top predictor with the highest SHAP values, particularly in LR, SVM, DT, and KNN models. This underscores the prognostic significance of renal function in predicting adverse outcomes in patients with MHT, a finding in line with previous publications.^3^, ^7^ Additionally, SBP at the first follow‐up visit emerged as a key feature in several models, further highlighting its relevance in risk stratification.^5^ The identification of baseline creatinine and SBP at the first follow‐up visit as important predictors underscores the importance of renal function and BP control in the management of patients with MHT. Our findings suggest that incorporating these variables into risk prediction models may enhance prognostication and guide clinical decision‐making, facilitating early interventions to mitigate adverse outcomes. Furthermore, basic demographic and cardiovascular risk factors, namely age and smoking, emerged as significant contributors to the models. In terms of the secondary endpoints, results were quite consistent as in the primary endpoint for the prediction of all‐cause death, with age, baseline creatinine, smoking, and SBP at the follow‐up visit as the main contributors. When considering dialysis, the presence of different indices of renal damage, such as hematuria and proteinuria, also emerged in LR, MLP and SVM models, together with alcohol consumption. However, the much smaller sample size in this subgroup may significantly affect the performance of the ML models. Taken all the above into consideration, our results indicate the need for a holistic and multidisciplinary approach in the management of MHT patients, as already advocated for various chronic long‐term conditions.^24^, ^25^, ^26^, ^27^

### 4.1 Strengths and Limitations

Despite the strengths of our study, including a relatively large sample size and comprehensive ML model evaluation, several limitations warrant consideration. The retrospective nature of the study and reliance on electronic health records may introduce bias and limit generalisability. Moreover, our data refer to a very specific population, which may not allow extrapolation to other settings. This specificity also constrains our models from conducting external validation in the present study design. Additionally, our analysis did not account for specific causes of mortality, a limitation stemming from the constraints of the original study design. We acknowledge the value of comparing ML approaches with established cardiovascular risk scores. However, MHT represents a distinct clinical entity with unique pathophysiology and complications that differ from general cardiovascular risk, and no validated specific prediction models exist for these patients. Additionally, this study is limited by the lack of precise timing for follow‐up blood pressure measurements, as they were recorded during subsequent clinical visits without standardised intervals. Lastly, given the significant advances in hypertension management in more recent years, the prognosis of MHT patients may have changed. Although this registry was established in 1977, follow‐up extended through 2016. This means that patients were managed over several decades, during which most of the currently available antihypertensive agents became widely used, along with more comprehensive strategies for cardiovascular disease management. We acknowledge, however, that caution is warranted when interpreting these findings in the context of contemporary clinical practice. Future studies incorporating prospective data collection and external validation are warranted to further assess the generalisability and robustness of our findings.

In conclusion, our study demonstrates the potential utility of ML models in predicting all‐cause mortality or dialysis requirements in patients with MHT. By leveraging key clinical variables, including baseline creatinine and systolic blood pressure, our models achieve robust predictive performance, offering valuable insights for risk stratification and clinical decision‐making in this high‐risk population.

## AUTHOR CONTRIBUTIONS

Antonios A. Argyris: Writing‐original draft, Conceptualization, Methodology. Hironori Ishiguchi: Formal analysis, Writing‐review and editing. Yang Chen: Formal analysis, Conceptualization, Methodology, Writing‐review and editing. Yalin Zheng: Writing – review and editing, Supervision. Alena Shantsila: Writing – review and editing, Supervision. Eduard Shantsila: Writing – review and editing, Supervision. Gregory Y. H. Lip: Data curation, Conceptualization, Methodology, Writing – review and editing, Supervision.

## FUNDING INFORMATION

Dr. Antonios Argyris was supported with a grant by the Hellenic Atherosclerosis Society.

## CONFLICT OF INTEREST STATEMENT

GYHL: Consultant and speaker for BMS/Pfizer, Boehringer Ingelheim, Daiichi‐Sankyo, Anthos. No fees are received personally. He is a National Institute for Health and Care Research (NIHR) Senior Investigator and co‐PI of the AFFIRMO project on multimorbidity in AF (grant agreement No. 899871), TARGET project on digital twins for personalized management of atrial fibrillation and stroke (grant agreement No. 101136244) and ARISTOTELES project on artificial intelligence for management of chronic long‐term conditions (grant agreement No. 101080189), which are all funded by the EU's Horizon Europe Research and Innovation programme. The rest of the authors declare no conflicts of interest related to this work.

## Supporting information


Appendix S1.

## Data Availability

The datasets analysed during the current study are not publicly available due to privacy and confidentiality agreements.
