# Supplementary material for: Prediction of major outcomes in patients with malignant hypertension using machine learning: A report from the West Birmingham malignant hypertension registry
Source: Eur J Clin Invest. 2025 Apr 18;55(9):e70052. doi: 10.1111/eci.70052 (PMC12362051; doi:10.1111/eci.70052)

**Supplement Figure S1.** Presentation of the flow diagram of the present study.


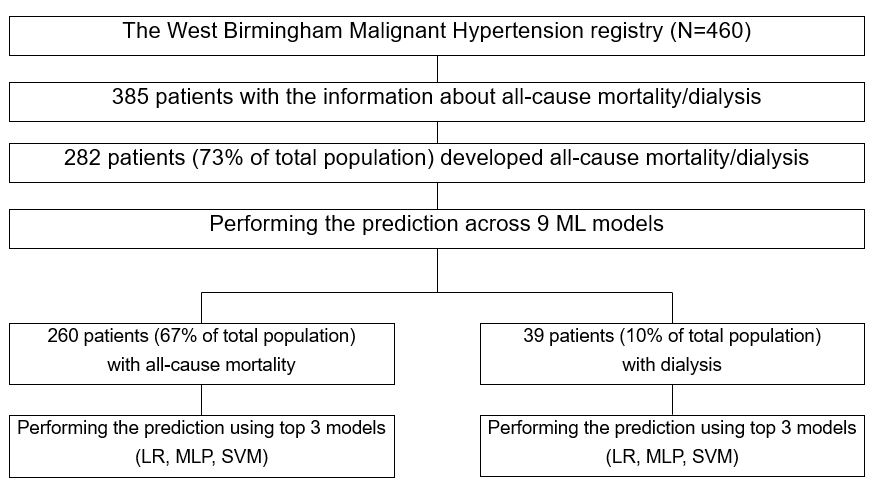


LR, logistic regression; ML, machine learning; MLP, multi-layer perceptron; SVM, support vector machine.

**Supplement Table S1.** Presentation of variance inflation factor (VIF) values across features used in models.

| **Features** | **VIF value** |
| --- | --- |
| Age (dichotomized by 48 years) | 2.34 |
| Male Sex | 3.50 |
| White ethnicity | 4.15 |
| Smoking status | 2.6 |
| Alcohol intake | 1.48 |
| Diabetes | 1.11 |
| Antihypertensive treatment | 1.42 |
| Retinopathy | 3.03 |
| Proteinuria | 3.08 |
| Hematuria | 1.45 |
| Baseline SBP (dichotomized by 228 mmHg) | 2.72 |
| Baseline DBP (dichotomized by 142 mmHg) | 2.35 |
| Na (dichotomized by 138 mmol/l) | 2.21 |
| K (dichotomized by 4.0 mmol/l) | 1.37 |
| FU1 SBP (dichotomized by 162 mmHg) | 2.05 |
| Baseline creatinine (µmol/l) | 2.11 |

DBP: diastolic blood pressure, FU1: first follow up visit, SBP: systolic blood pressure.

**Supplement Figure S2.** Presentation of the correlation coefficients between variables.


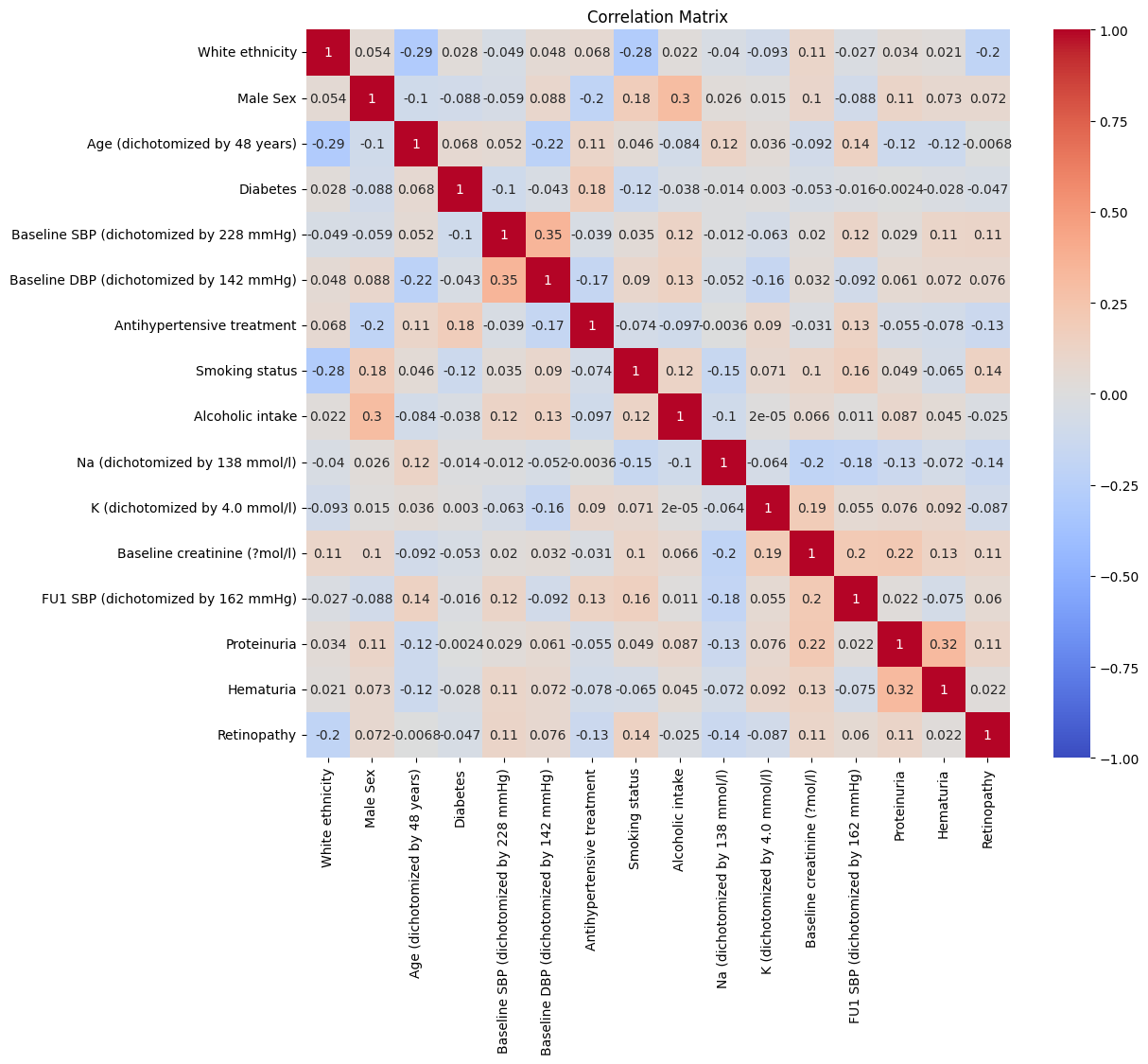


Each value indicated the correlation coefficients with other variables.

DBP: diastolic blood pressure, FU1: first follow up visit, FU2: second follow up, PP: pulse pressure, SBP: systolic blood pressure.

**Supplement Table S2.** Hyperparameter values in each model for the primary outcome.

| **Model** | **Hyperparameter** | **Value** |
| --- | --- | --- |
| LR | C | 1 |
|  | Penalty | L1 |
|  | Solver | bilinear |
| MLP | Hidden layer sizes | (100,50) |
|  | Activation | tanh |
|  | Solver | adam |
|  | Alpha | 0.001 |
|  | Learning rate | constant |
| SVM | C | 100 |
|  | Kernel | rbf |
|  | Degree | 4 |
|  | Gamma | scale |
| RF | N estimators | 200 |
|  | Max depth | None |
|  | Min samples split | 10 |
|  | Min samples leaf | 4 |
| CatBoost | Iteration | 50 |
|  | Depth | 8 |
|  | Learning rate | 0.1 |
|  | 12 leaf reg | 1 |
|  | Border count | 64 |
| LightGBM | N estimators | 50 |
|  | Num leaves | 31 |
|  | Max depth | -1 |
|  | Learning rate | 0.05 |
|  | Subsample | 0.8 |
| XGBoost | N estimators | 100 |
|  | Max depth | 4 |
|  | Learning rate | 0.1 |
|  | Reg lambda | 1.5 |
|  | Booster | gbtree |
| KNN | N neighbours | 7 |
|  | Weights | distance |
|  | Algorithm | ball tree |
|  | Leaf size | 10 |
|  | P | 1 |
| DT | Max depth | None |
|  | Min samples split | 20 |
|  | Min samples leaf | 8 |
|  | max features | sqrt |

DT: decision tree, GBM: gradient boosting machine, KNN: K-nearest neighbours, LR: logistic regression, MLP: multi-layer perceptron, RF: random forest, SVM: support vector machine.

**Supplement Figure S3**. Presentation of key factors and their contribution for primary endpoint in different machine learning models: Each plot was organized by importance, with the top indicating the highest contribution. i) logistic regression (LR), ii) multi-layer perceptron (MLP), iii) support vector machine (SVM), iv) Light gradient boosting machine (LightGBM), v) random forest (RF), vi) CatBoost, vii) XGBoost, viii) decision tree (DT), ix) K-nearest neighbors (KNN).

i)


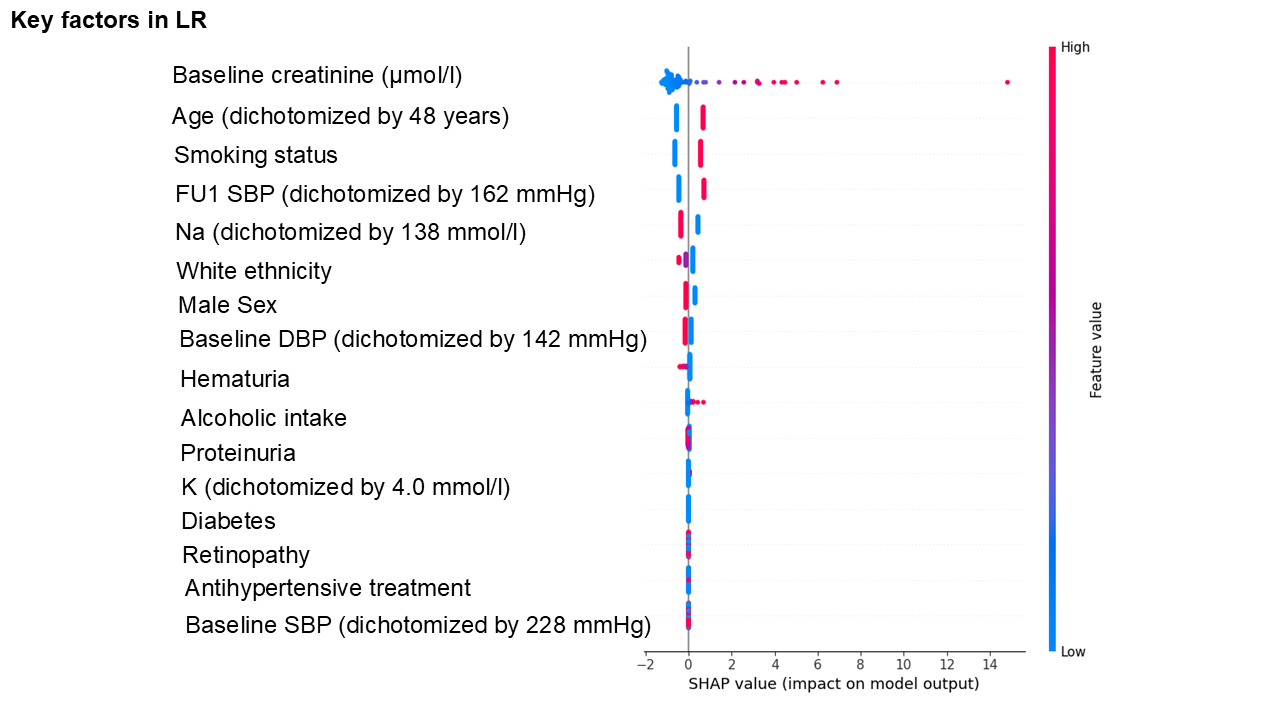


ii)


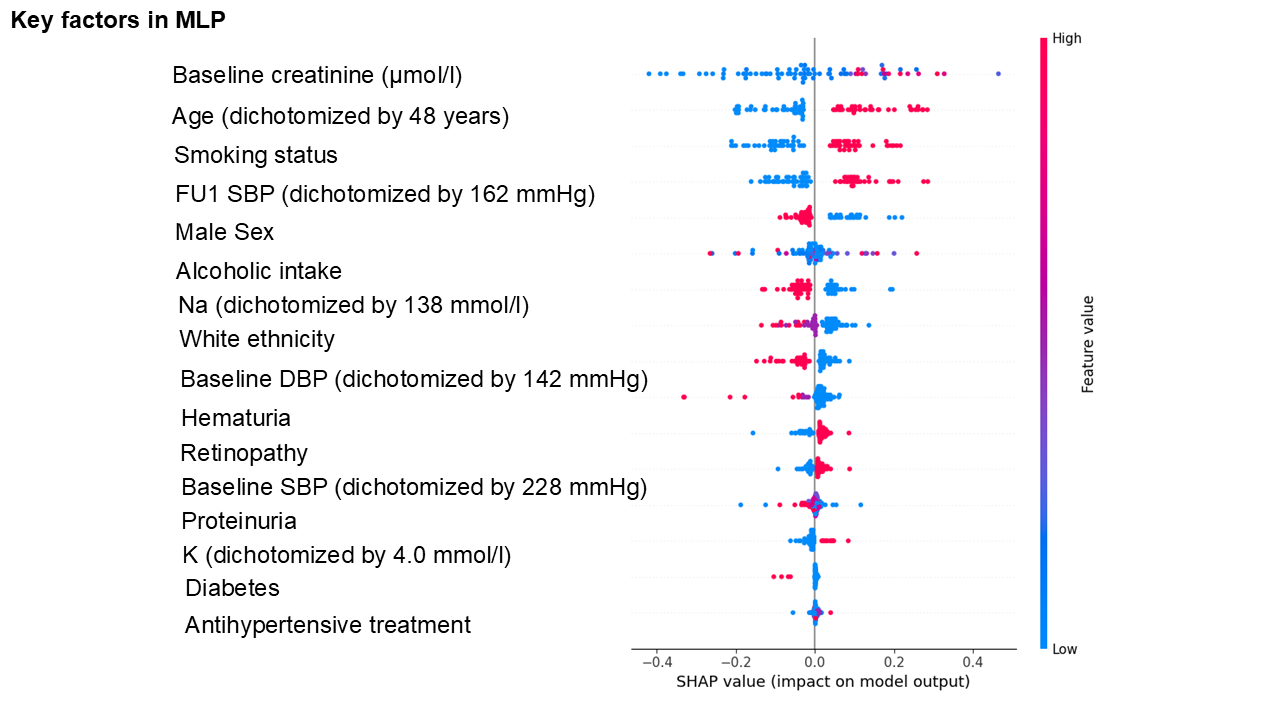


iii)


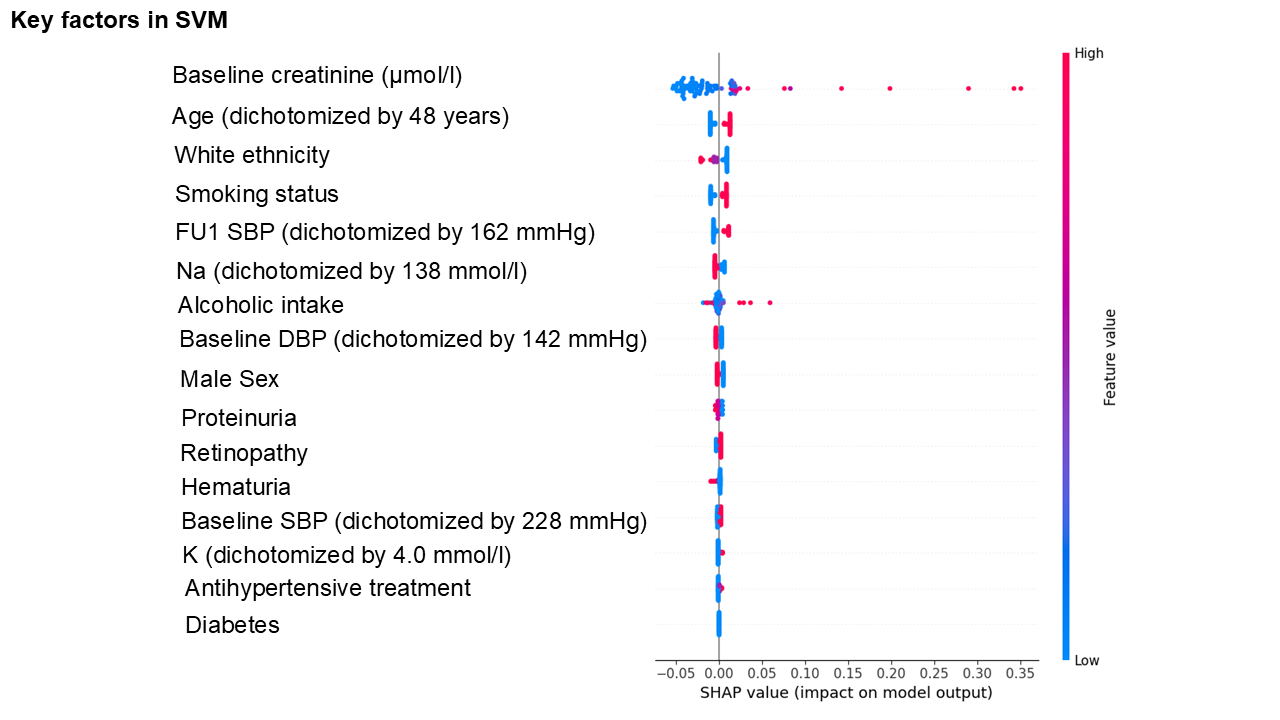


iv)


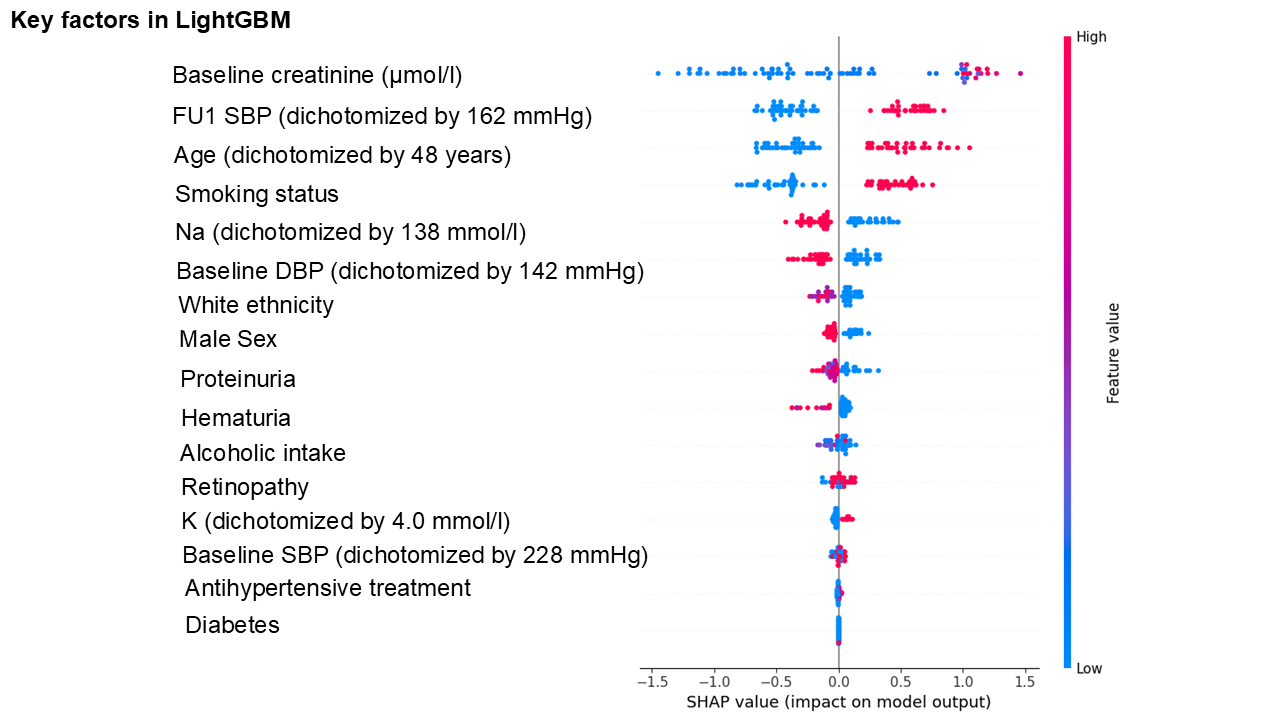


v)


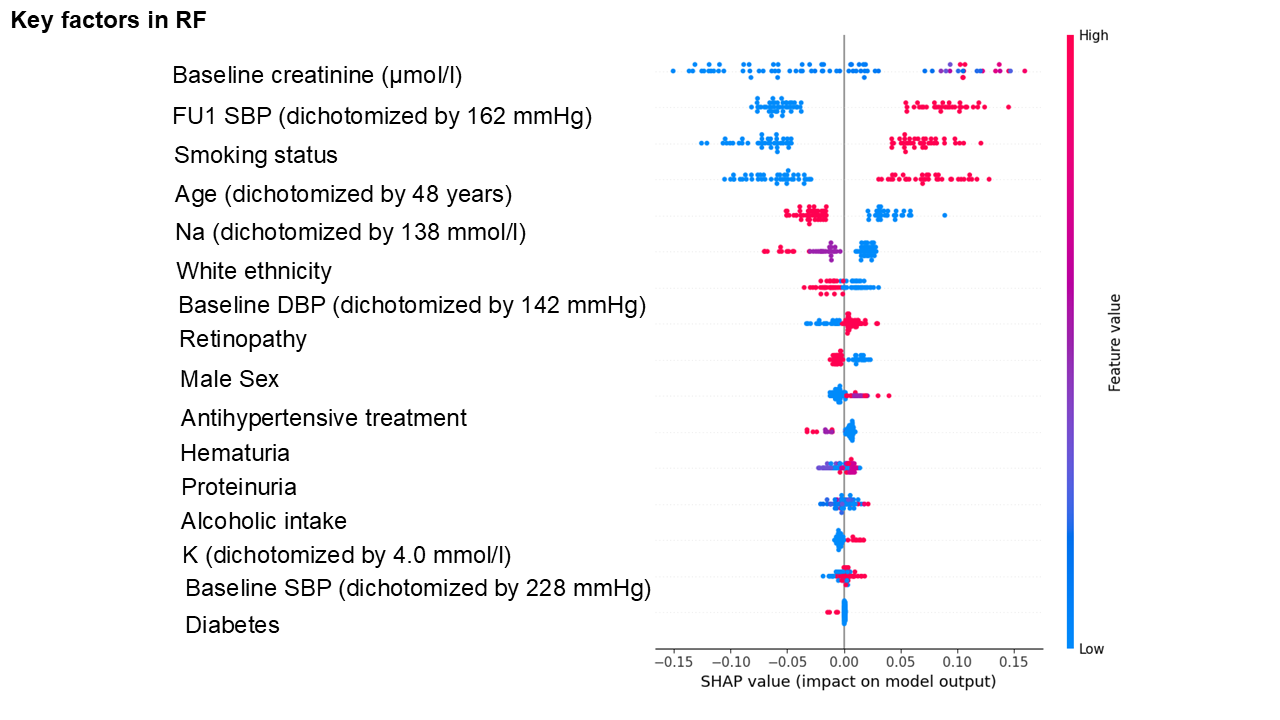


vi)


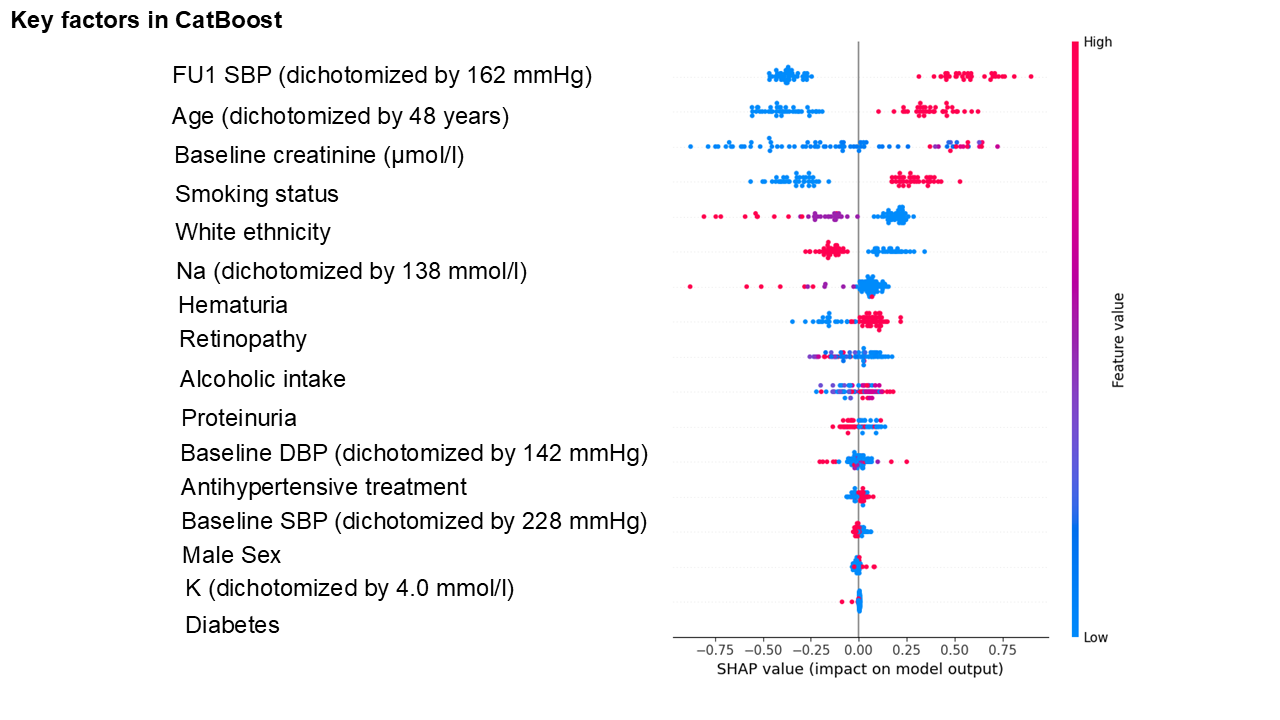


vii)


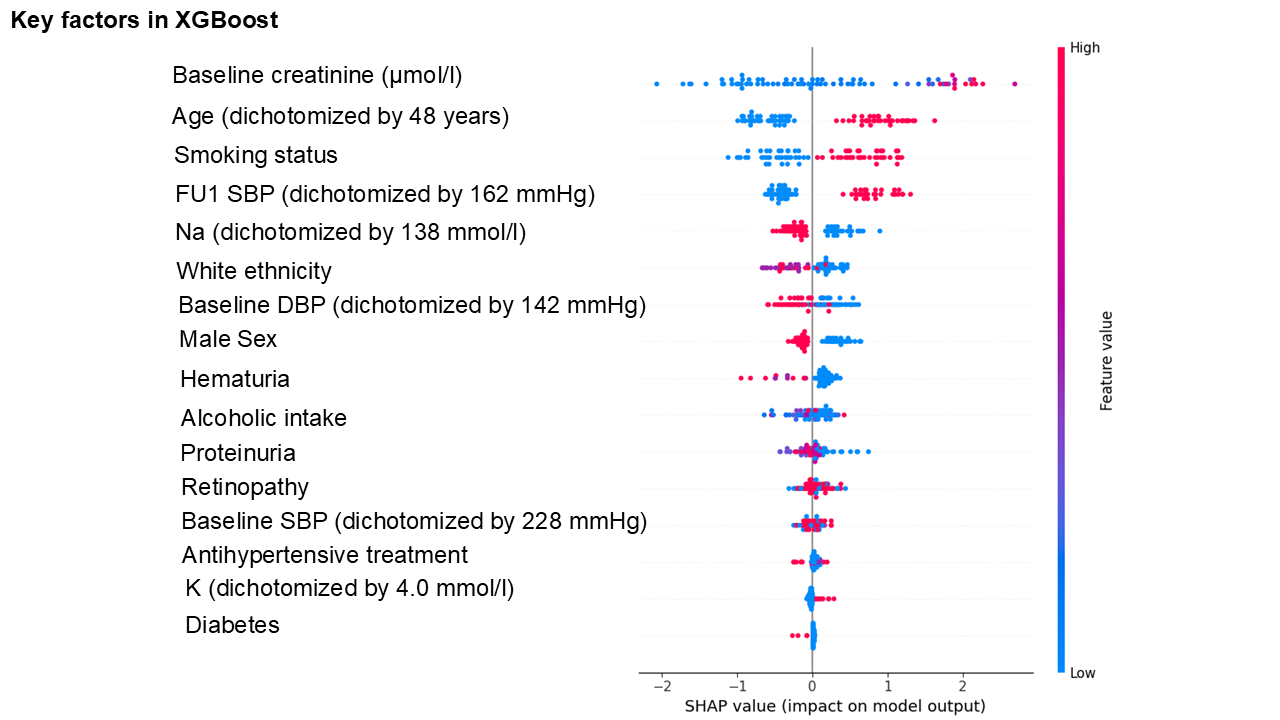


viii)


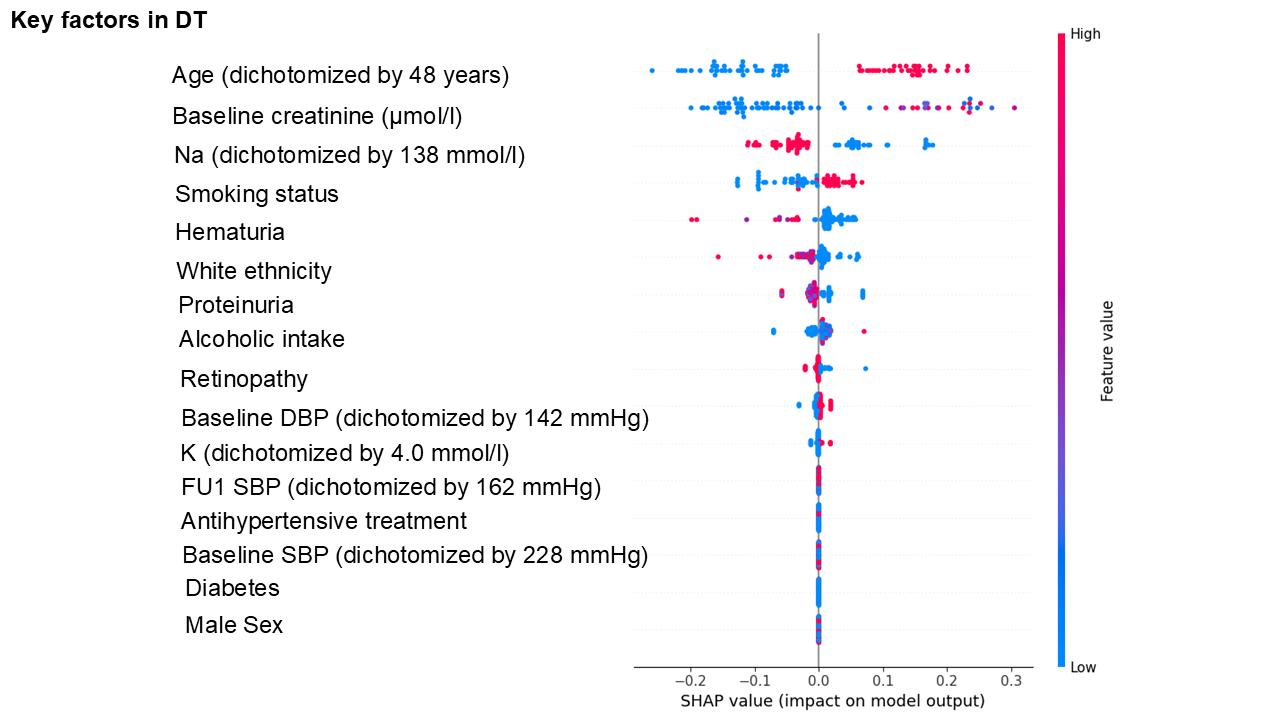


ix)


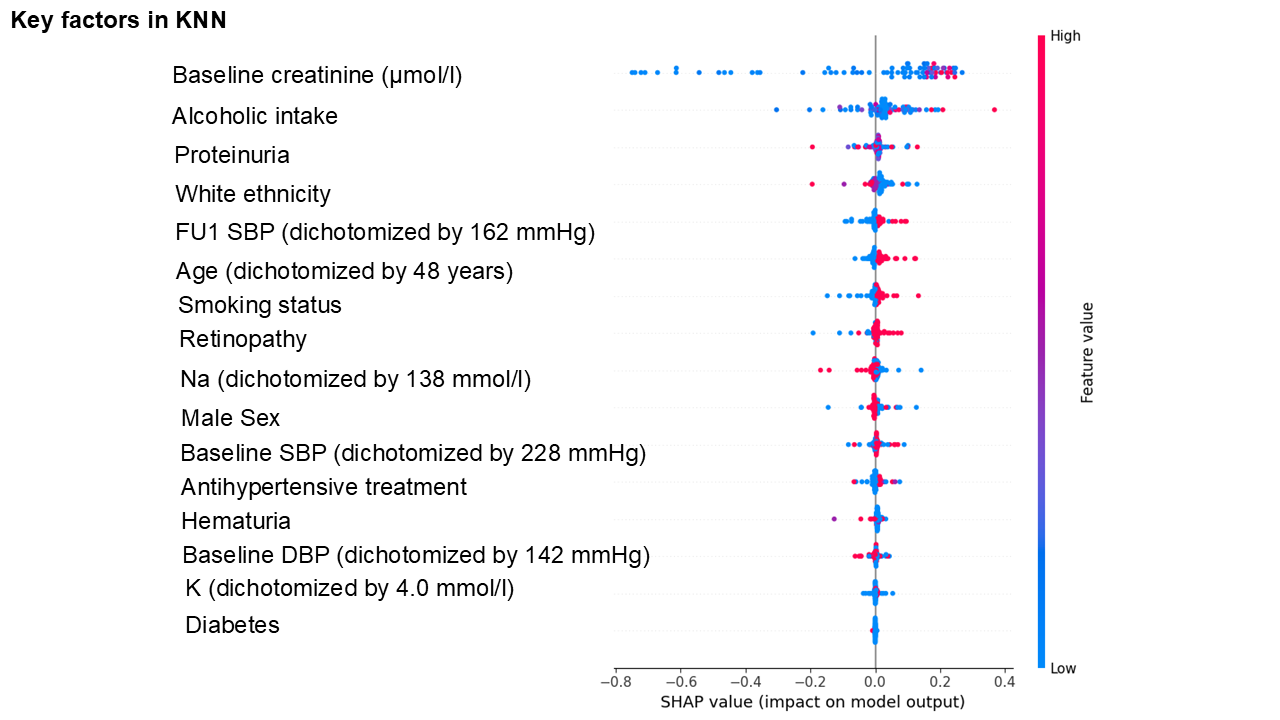


**Supplement Table S3.** Hyperparameter values for secondary endpoints in each model.

| **Model** | **Hyperparameter** | **Value** |
| --- | --- | --- |
| **For all-cause mortality** | | |
| LR | C | 1 |
|  | Penalty | L1 |
|  | Solver | bilinear |
| MLP | Hidden layer sizes | (100,50) |
|  | Activation | identity |
|  | Solver | lbfgs |
|  | Alpha | 0.001 |
|  | Learning rate | constant |
| SVM | C | 100 |
|  | Kernel | rbf |
|  | Degree | 4 |
|  | Gamma | scale |
| **For dialysis** | | |
| LR | C | 0.1 |
|  | Penalty | L1 |
|  | Solver | bilinear |
|  | Class weight | balanced |
| MLP | Hidden layer sizes | (100,50) |
|  | Activation | identity |
|  | Solver | lbfgs |
|  | Alpha | 0.001 |
|  | Learning rate | constant |
| SVM | C | 100 |
|  | Kernel | rbf |
|  | Degree | 4 |
|  | Gamma | scale |

LR: logistic regression, MLP: multi-layer perceptron, SVM: support vector machine.

**Supplement Figure S4**. Presentation of key factors and their contribution for secondary endpoint (all-cause mortality) in different machine learning models: Each plot was organized by importance, with the top indicating the highest contribution. i) logistic regression (LR), ii) multi-layer perceptron (MLP), iii) support vector machine (SVM).

i)


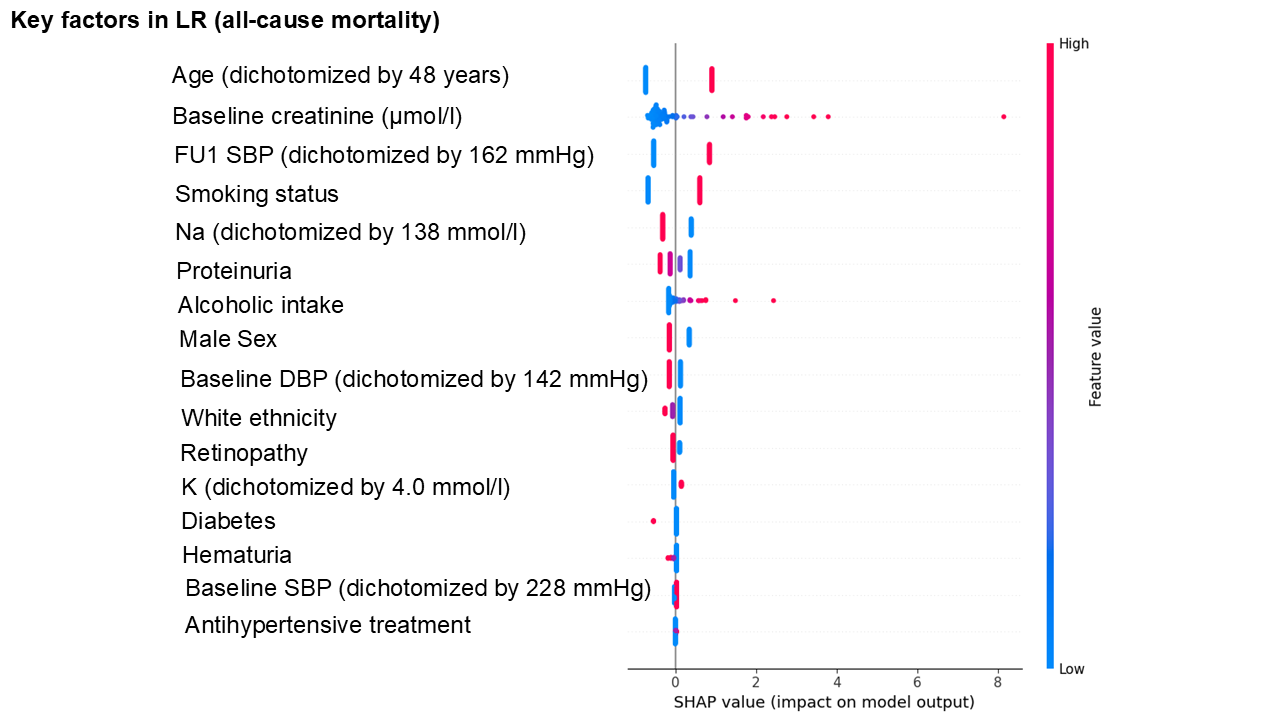


ii)


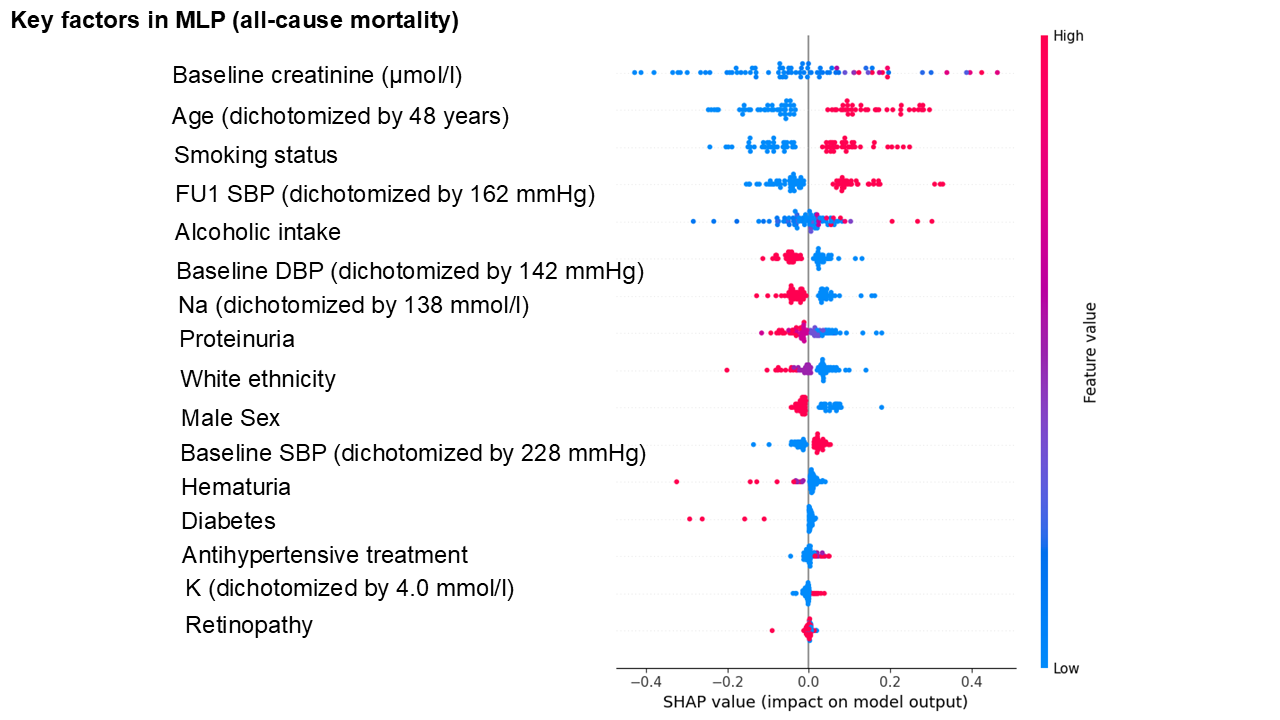


iii)


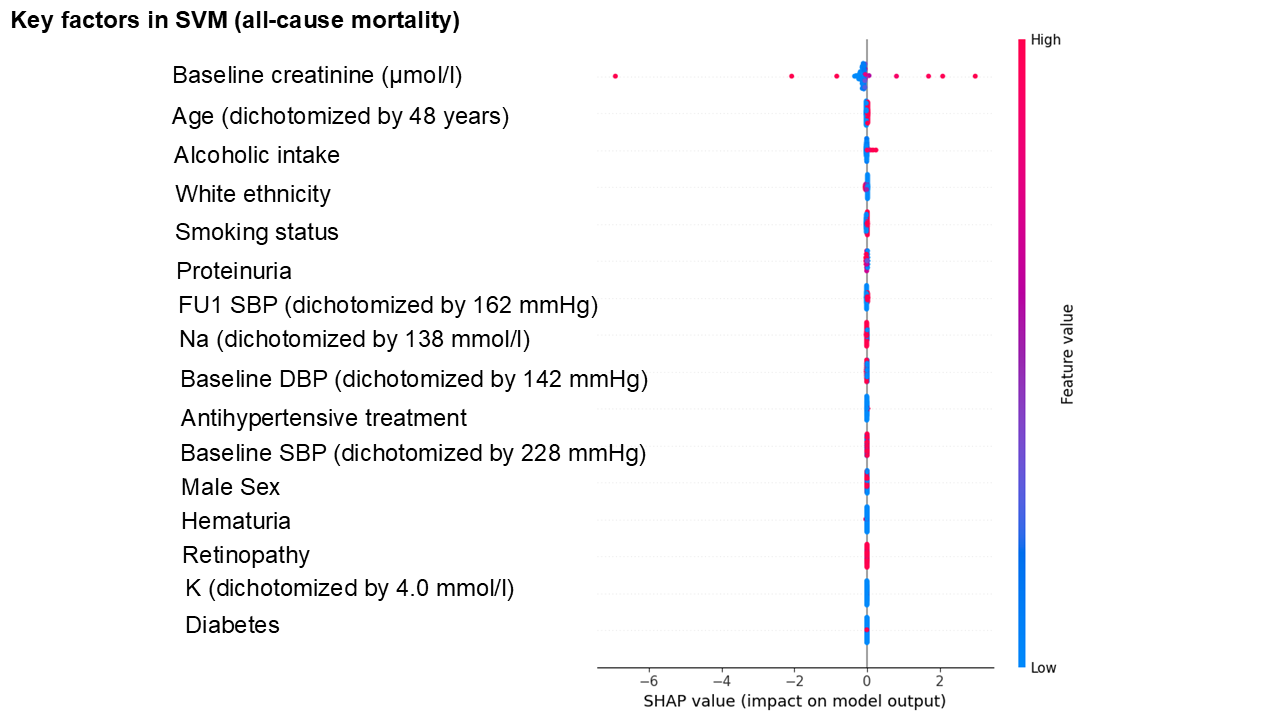


**Supplement Figure S5**. Presentation of key factors and their contribution for secondary endpoint (dialysis) in different machine learning models: Each plot was organized by importance, with the top indicating the highest contribution. i) logistic regression (LR), ii) multi-layer perceptron (MLP), iii) support vector machine (SVM).

i)


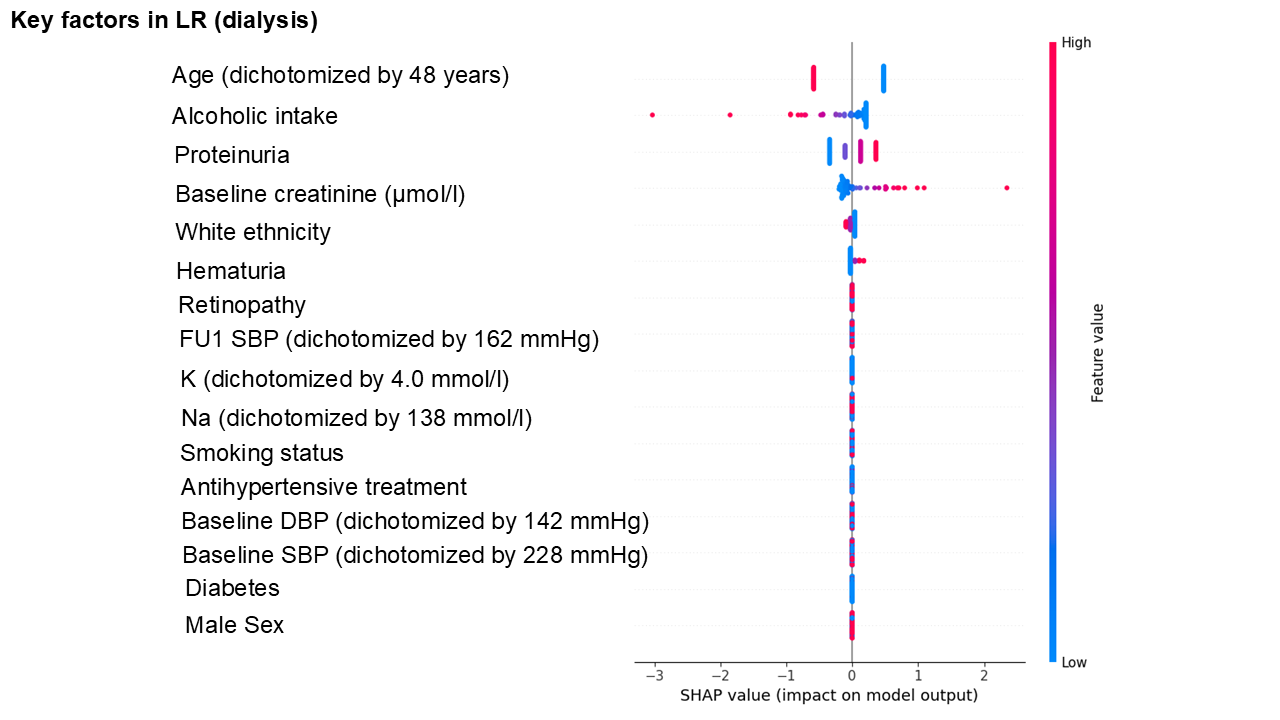


ii)


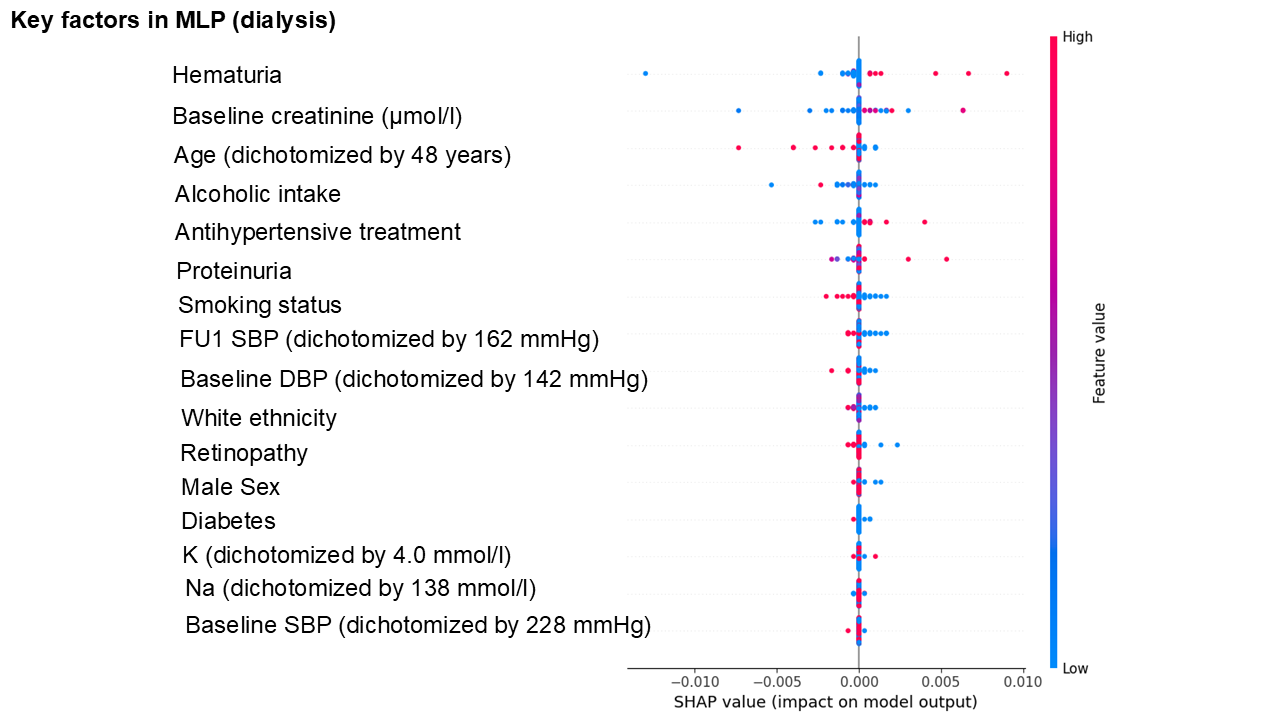


iii)


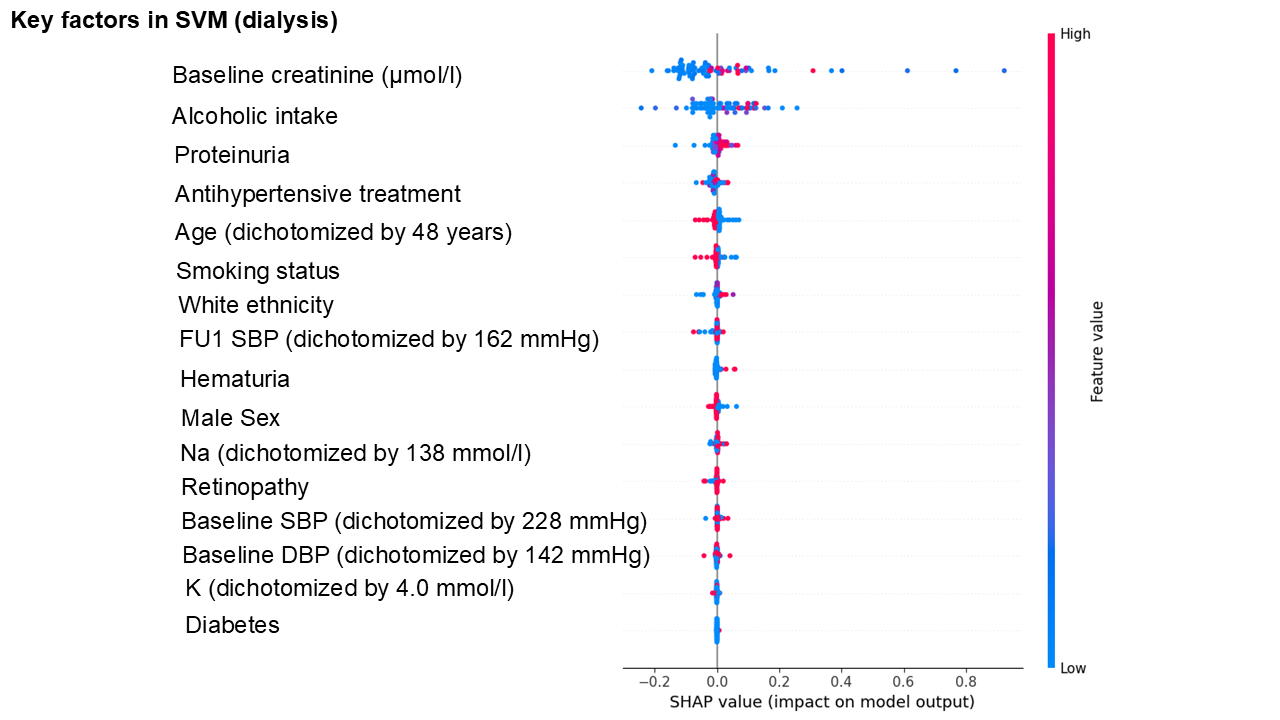

Supplement: Supplementary file 1 — Appendix S1. [file ECI-55-e70052-s001.docx]
